# *Ssams2*, a Gene Encoding GATA Transcription Factor, Is Required for Appressoria Formation and Chromosome Segregation in *Sclerotinia sclerotiorum*

**DOI:** 10.3389/fmicb.2018.03031

**Published:** 2018-12-06

**Authors:** Ling Liu, Qiaochu Wang, Xianghui Zhang, Jinliang Liu, Yanhua Zhang, Hongyu Pan

**Affiliations:** College of Plant Sciences, Jilin University, Changchun, China

**Keywords:** RNA interference, *Sclerotinia sclerotiorum*, SsAMS2, appressoria, virulence, chromosome segregation

## Abstract

AMS2, a multicopy suppressor for the *cpn1* (SpCENP-A) mutant, functions to specifically regulate histone genes transcription and chromosome segregation. As a cell-cycle-regulated GATA transcription factor in eukaryotic organisms, little research has been done on the role of AMS2 protein in pathogenic fungi. In *Sclerotinia sclerotiorum*, *Ssams2* (SS1G_03252) encodes a protein which has been predicted to contain GATA-box domain. Here, *Ssams2*-silenced strains with significantly reduced *Ssams2* gene expression levels exhibited defect in hyphal growth, hyphal branching patterns, compound appressoria differentiation and the oxalic acid production compared to the wild-type (WT) strain. By common bean leaves infection assays, we identified the role of *Ssams2* in full virulence. Furthermore, the numbers of cell nucleus in the same length of mycelium in *Ssams2*-silenced transformants were significantly less than that in the WT strain. The expression levels of histone genes and cell cycle genes in transformants were down-regulated significantly in the RNAi strains. Taken together, our work suggests that the TF SsAMS2 is required for growth, appressoria formation, virulence, and chromosome segregation in *S. sclerotiorum*.

## Introduction

The Leotiomycetes fungus *Sclerotinia sclerotiorum* (Lib.) de Bary is a notorious plant pathogen, it infects over 600 plant species and incites rapid host tissue maceration in a non-discriminant manner (Liang and Rollins, 2018). Economical crops such as rape, soybean, sunflower, lettuce can be infected by *S. sclerotiorum* (Boland and Hall, 1994). As a host-nonspecific fungal pathogens, *Sclerotinia* diseases showed the limited tissue specificity and spreading necrosis (Rollins, 2003). *S. sclerotiorum* lacks of mitotic spores (Jurick Ii et al., 2004), but produces hardened, multicellular, highly melanized sclerotia which are capable survival for several years in hostile environment, including dry environments, low temperature, acidity, basicity, or microbial active soils (Bolton et al., 2006; Li et al., 2017). Sclerotia as a crucial structure in survivability and pathogenicity of *S. sclerotiorum* can differentiate either into vegetative hyphae or into apothecia which could release a lot of airborne ascospores to infect host plant and start a new disease cycle (Steadman, 1979; Wang et al., 2016; Fan et al., 2017).

*S. sclerotiorum* as a necrotrophic ascomycete can infect a broad range of plant species (Lecompte et al., 2013). To effectively infect plants the *S. sclerotiorum* have evolved sophisticated infection process (Kabbage et al., 2015). The compound appressorium (infection cushions) adhere to the host surface and penetrate the healthy plant cuticle (Uloth et al., 2016; Liang and Rollins, 2018). Infection cushions were the multicellular, melanin-rich hyphal penetration structure (Li et al., 2017). When contact with host, the hyphae of *S. sclerotiorum* become extensively branched, exhibit hook and bifurcate (Lumsden and Dow, 1973; Tariq and Jeffries, 1984; Jamaux et al., 2007; Huang et al., 2008; Xiao et al., 2014). Sticky mucilage around the infection cushions increase the adhesion of *S. sclerotiorum* hyphae (Tariq and Jeffries, 1984), and thin penetration pegs originate from compound appressorium can perforate plant cuticle (Huang et al., 2008; Xiao et al., 2014). Oxalic acid (OA) as a non-host-selective toxin, play very important roles in phytopathogenic fungi (Liang et al., 2015a; Xu et al., 2015). In *S. sclerotiorum*, OA is released and macerated the host tissues, help the pathogen kill the host (Andrew et al., 2012; Heller and Witt-Geiges, 2013). Therefore, if the hyphae growth, OA production or the appressorium differentiation were blocked, *Sclerotinia* diseases could be controlled effectively.

GATA type zinc finger transcription factor (TF) was widely found in eukaryotes, plays critical roles in regulating physiological metabolism and controlling multicellular development (Lowry and Atchley, 2000; Liu et al., 2018). The *ams2* gene, a multicopy suppressor for the *cpn1* mutant in fission yeast (Chen et al., 2003), encodes a GATA factor, binds to the histone gene promoter regions and takes part in transcriptional activation of the histone genes (Takayama et al., 2016). In *Schizosaccharomyces pombe*, AMS2, assembled in promoter regions of putative target genes and enriched in central centromeres, is critical for chromosome segregation, possibly via regulating localization of SpCENP-A (*cpn1*) which is the centromere-specific histone H3 variant (Takayama et al., 2008). In S phase of cell cycle, an amount of histone is synthesized and appropriate histone gene expression levels are essential for chromosome segregation and genome integrity (Gunjan et al., 2005). The expression of *ams2* comes to the peak in S phase and the deletion or overproduction of *ams2* affects the normal synthesis of histones (Takayama et al., 2010). In *ams2*-deficient cell, the division cycle of the mutant cell is longer and the histone genes accumulation were markedly reduced during DNA replication (Takayama and Takahashi, 2007). Overproduction of AMS2 affect the normal meiosis process, histone deposition into centromeres and genetic stability (Trickey et al., 2013). The cell cycle relevant *ams2* expression level is important to the centromere nucleosome formation and genetic stability.

Because AMS2 protein is vital to chromosome segregation and play a role in the cell division, we closely examine the SsAMS2 protein as a *S. pombe* AMS2 homolog in *S. sclerotiorum.* We probed the *S. sclerotiorum* genome and used RNAi strategy to down-regulate the *Ssams2* gene expression. The results showed that SsAMS2 protein was involved in hyphal growth, appressoria formation, and chromosome segregation.

## Results

### Identification and Sequence Analysis of *Ssams2*

**FIGURE 1:**
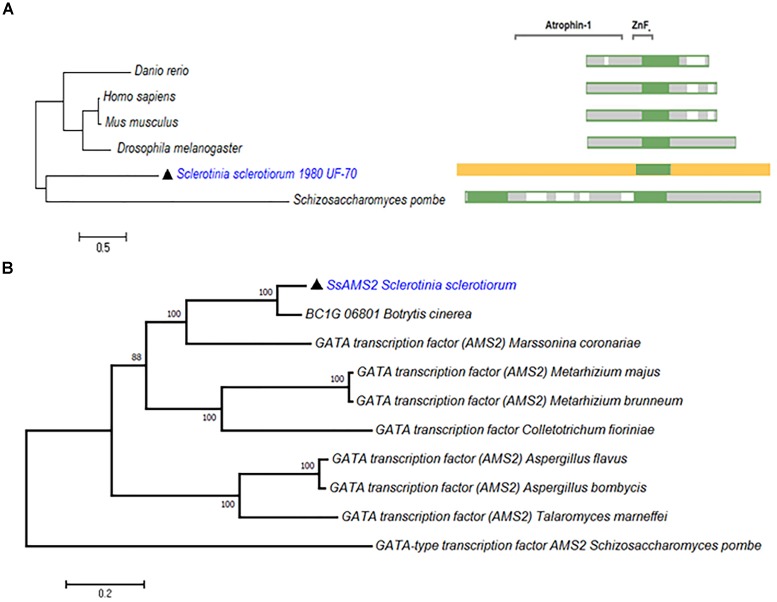
SsAMS2 is a GATA-TF and highly conserved among fungi. **(A)** SsAMS2 sequence was used to SmartBLAST with NCBI, the query sequences by *Danio rerio*, *Mus musculus*, *Homo sapiens*, *Drosophila melanogaster*, and *Schizosaccharomyces pombe* were choosed, white color represents the gaps, green color represents landmark matches. **(B)** Phylogenetic analysis of *S. sclerotiorum* SsAMS2 and other homologous GATA transcription factors from *Botrytis cinerea*, *Marssonina coronariae*, *Metarhizium brunneum*, *Metarhizium majus*, *Colletotrichum fioriniae*, *Aspergillus flavus*, *Aspergillus bombycis*, *Talaromyces marneffei*, *Schizosaccharomyces pombe*, SsAMS2 marked with 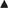.

From the genome of *S. sclerotiorum*, nine proteins containing GATA-domains are predicted based on PFAM (Amselem et al., 2011; Liu et al., 2018). Among them, a gene (SS1G_03252) which encode a protein contains 1201 amino acids. Phylogenetic analysis demonstrate that SsAMS2 homologous proteins can be found in *Homo sapiens*, *Danio rerio*, *Drosophila melanogaster*, *Mus musculus*, and *S. pombe* (Figure 1A). Sequence alignment of SsAMS2 was performed and phylogenetic analysis using different fungi species, such as *Marssonina coronariae*, *Metarhizium brunneum*, *Botrytis cinerea*, *Metarhizium majus*, *Colletotrichum fioriniae*, *Aspergillus flavus*, *Aspergillus bombycis*, *Talaromyces marneffei*, and *S. pombe*. The results displayed that GATA box-containing protein (SS1G_03252) is classed with the AMS2 TF (Figures 1A,B). Taken together the results from the phylogenetic analysis, the choosed protein in *S. sclerotiorum* was named as SsAMS2. To examine the expression patterns of *Ssams2* during hyphae, sclerotial and apothecium developmental stages in *S. sclerotiorum*, qRT-PCR was used to test the expression levels of *Ssams2*. The results indicate that the *Ssams2* was down-regulated expression during the sclerotia and apothecium stages (Supplementary Figure S1).

### RNAi-Mediated Down-Regulation of *Ssams2* Impairs Mycelium Growth

**FIGURE 2:**
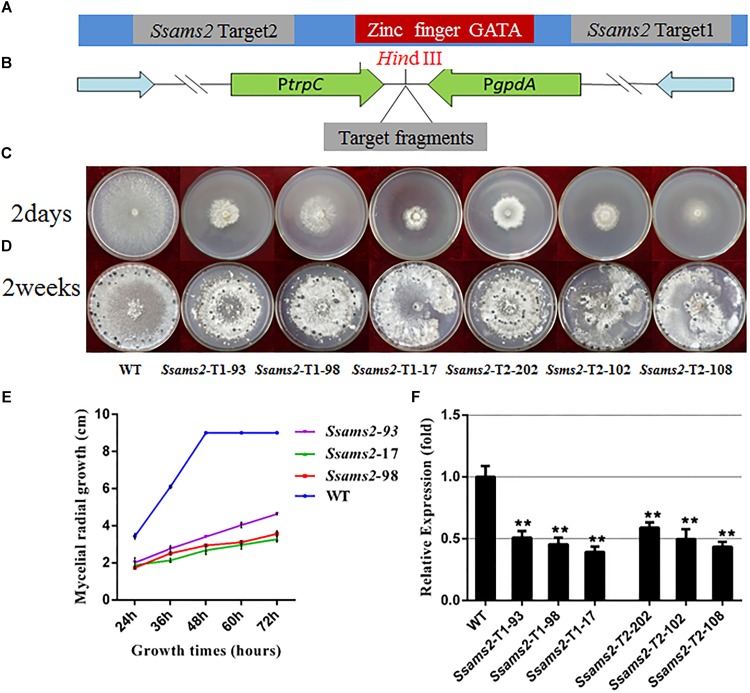
Morphologic observation of the *Ssams2*-silenced strains. **(A,B)** The silence vectors construction of *Ssams2* gene in *S. sclerotiorum*. **(C)** Mycelium growth of WT, *Ssams2*-Target 1-silenced strains and *Ssams2*-Target 2-silenced strains. **(D)** Sclerotial morphology after 2 weeks of growth. **(E)** Hyphal growth rates of the RNAi strains and the WT strain cultured on PDA plates. **(F)** The *Ssams2* gene expression level was verified by qRT-PCR (one-way ANOVA, ^∗∗^indicates significance at *p* < 0.01).

To determine whether SsAMS2 influences the mycelium development of *S. sclerotiorum*, the *Ssams2* knockdown transformants were obtained. *S. sclerotiorum* is a multi-nucleated fungus and RNA silencing is a mature gene function research strategy in fungi (Lyu et al., 2016; Liu et al., 2018). The result of sequence alignment (Blastn) showed that the *Ssams2* gene sequence was highly specific (Supplementary Figure S2). Two *Ssams2* target fragments which have no overlapping and outside of GATA zinc finger domain were designed (Figure 2A). The Target 1 (254 bp) and Target 2 (352 bp) fragments were ligated into the pSilent-Dual 1 (pSD1) vector (Nguyen et al., 2008; Fan et al., 2017; Liu et al., 2018; Figure 2B). The resulting constructs (Figures 2A,B) were used to transform the *S. sclerotiorum* protoplast (Rollins, 2003; Qu et al., 2014). The transformants were selected on PDA medium with geneticin and confirmed through the amplification of the geneticin resistance gene (Supplementary Figure S3). Six transformants were designated as *Ssams2*-T1-93, *Ssams2*-T1-98, *Ssams2*-T1-17 (originated from Target 1 transformation) and *Ssams2*-T2-202, *Ssams*2-T2-102, *Ssams2*-T2-108 (originated from Target 2 transformation). The *Ssams2* transformed strains obtained from the two targets showed a similar phenotype and we can trust the accuracy of the RNAi strategy (Lyu et al., 2015; Liu et al., 2018).

**FIGURE 3:**
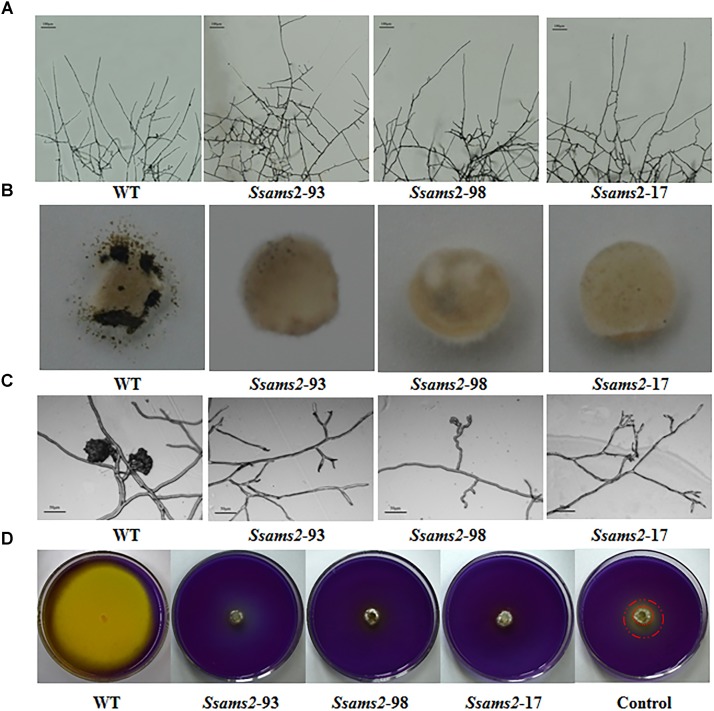
Infection cushions development and OA production assayed. **(A)** The observation of mycelium branches from the RNAi strains and the WT strain. The photographs were taken after 16 h with a light microscope. Scale bars represent 100 μm. **(B)** Infection cushions production of WT strain and *Ssams2*-silenced strains. Mycelia plugs were inoculating on glass slid and the pictures were obtained after 3 dpi (5 mm in diameter). **(C)** Infection cushions on cellophane overlaid PDA were observed by light microscopy 16 hpi. Scale bars represent 50 μm. **(D)** WT and *Ssams2*-silenced strains were assayed for OA production on PDA medium supplemented with bromophenol blue. The control was other mutant which mycelium grows slowly but does not affect oxalic acid production.

SsAMS2 is homologous to protein AMS2, which has been demonstrated to be involved in activation the core histone genes expression at S phase of cell cycle in *S. pombe* (Takayama et al., 2016). In *ams2*-deficient cell, the division cycle is longer than that in wild-type cells (Takayama and Takahashi, 2007). Therefore, we hypothesized that SsAMS2 was involved in hyphal development in *S. sclerotiorum*. The RNAi transformants exhibited defect in mycelial growth. Both the Target 1 transformants and the Target 2 transformants exhibited impaired growth phenotypes (Figures 2C,D). The mycelium colony diameter of RNAi strains was significantly lower than that of WT, *Ssams2* gene was down-regulate expression and the cell growth rate was reduced (Figure 2E). qRT-PCR was used to verify the expression of *Ssams2* in different strains. The results showed that the accumulation of *Ssams2* gene at mRNA level were suppressed in different degrees (Figure 2F). Taken together, we found the similar phenotype within the Target 1 and the Target 2 transformants, so the Target 1 transformants were choosed for the subsequential experiments. The hyphal micro-morphology was observed under light microscope and found the hyphal branching patterns of *Ssams2*-silenced strains were perpendicular and short, whereas the wild-type strains were acute angle branching (Figure 3A). The results showed that *Ssams2* is associated with hyphal growth and hyphal branching patterns in *S. sclerotiorum*. The RNAi strains demonstrate abnormal sclerotial development. With the change of mycelium growth morphology, the number of sclerotia decreased and the distribution was different (Figure 2D and Supplementary Figure S4).

### *Ssams2* Is Involved in the Virulence of *S. sclerotiorum*

We have showed that reduced the expression of *Ssams2* could impaired the hyphal growth and hyphal branching patterns (Figures 2, 3A). In order to verify that *Ssams2* take part in the formation and development of compound appressoria (infection cushions), the experiment to induce the generation of compound appressoria was completed. In this assay, *Ssams2*-silenced transformants failed to produce infection cushions on glass slide. While, the WT strain had already produced mature infection cushion structures once contact with glass slide (Figure 3B). Mycelial plugs were inoculated on PDA medium covered with cellophane and microscopic observation revealed that the hyphal tips of RNAi transformants exhibited hook and bifurcate which like the initiation of compound appressorium. However, the hook and bifurcate did not develop into multihyphal appressoria or infection cushions (Figure 3C). WT, *Ssams2*-silenced strains and a mutant strain which affect hyphal growth but does not affect oxalic acid secretion (obtained and kept in our lab) were inoculated on PDA medium supplemented with bromophenol blue (BPB). The result confirmed that *Ssams2*-silenced transformants showed the OA-minus phenotype (Figure 3D and Supplementary Figure S4).

**FIGURE 4:**
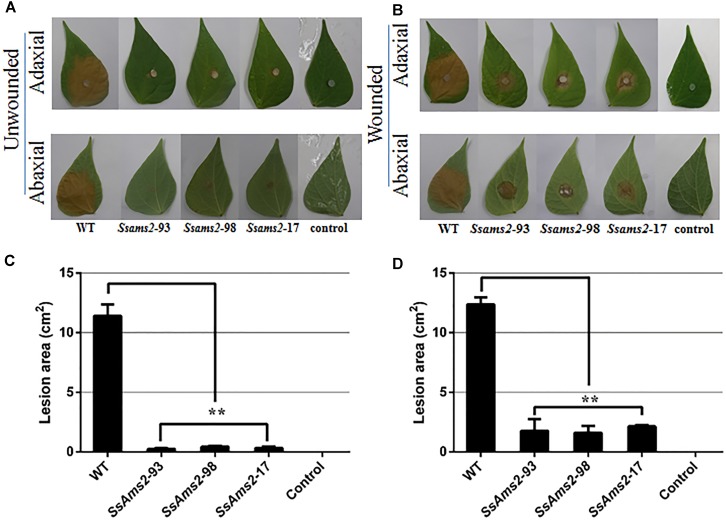
The absence of *Ssams2* gene affects full virulence of *S. sclerotiorum*. **(A,B)** *Ssams2*-silenced strains produced relatively small spots on the host leaves at 36 hpi (**A**: unwounded leaves; **B**: wounded leaves). The control was medium plug without fungi. The experiment was repeated three times. **(C,D)** The statistic analysis of the lesion size in panels **(A,B)**. (^∗∗^indicates significance at *p* < 0.01).

To examine how SsAMS2 is involved in process of pathogenic in *S. sclerotiorum*, the detached common bean leaves were inoculated with the agar plugs derived from WT strain and *Ssams2*-silenced transformants. The results show that the RNAi strains infection capacity were highly impaired in the infection process (Figures 4A,C). The observation that genetic mutants defective in compound appressorium formation exhibit virulence defects that can be rescued by wounding the host (Li et al., 2012). To monitor the penetration rescued events, host tissues were wounded then inoculated with the agar plugs and placed to the same condition as to the healthy leaves. RNAi strains distinct primary lesions emerged later than the WT strain. The area of the wounded host leaf colonized by RNAi strains was smaller than WT strain but the bigger necrotic lesions compared to the unwounded leaf (Figures 4B,D). These results suggested that virulence defects that can be partial rescued by wounding the host, the absence of *Ssams2* affect the production of compound appressorium and reduced penetration rates in *S. sclerotiorum*.

### Chromosome Segregation Is Disturbed in *Ssams2*-Silenced Strains

**FIGURE 5:**
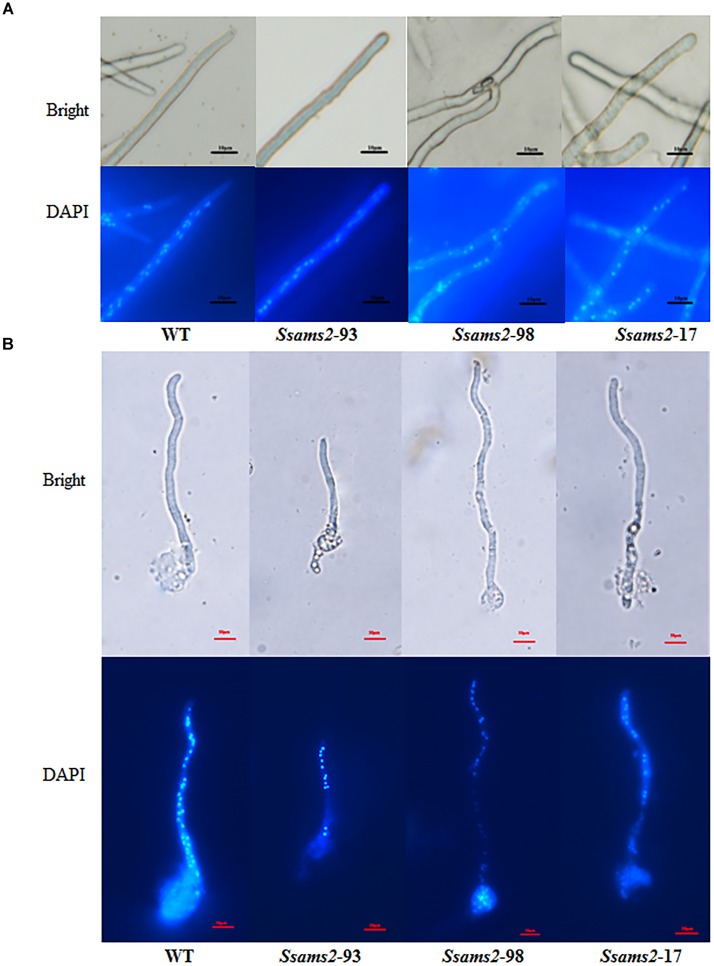
Cell nucleus observation in hyphae of *Ssams2*-silenced transformants and WT strain. Hyphae **(A)** and the germinating protoplasts **(B)** were stained by 4′,6-diamidino-2-phenylindole (DAPI) in the dark for 30 min and observed in fluorescence microscope. Scale bar represent 10 μm.

**FIGURE 6:**
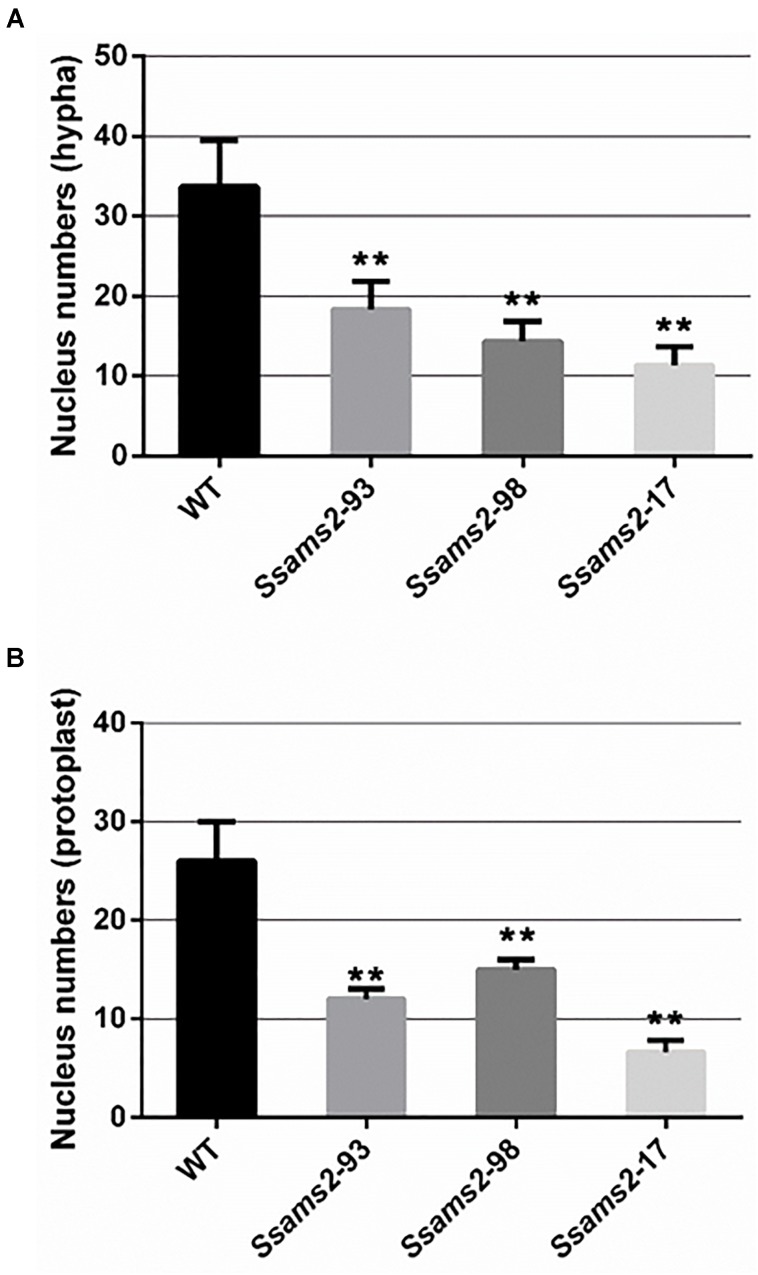
The numbers of cell nucleus were counted in hyphae of *Ssams2*-silenced transformants and WT strains. Count the numbers of cell nucleus in the same length of mycelium **(A)** and germinating protoplast **(B)**. Values represent the means ± standard deviation (SD) (*n* = 3). Asterisks indicate significant difference compared with the WT strain [^∗∗^*p*-value < 0.01, one-way analysis of variance (ANOVA)].

In fission yeast, overexpression of *ams2* could suppress the chromosome missegregation phenotype (Lim et al., 2015). To verify the role of *Ssams2* in the chromosome segregation phenotype, 4′,6-diamidino-2-phenylindole (DAPI) dye was used for routine nuclear staining, cell nucleus in hyphae and germinating protoplasts (PDA) were observed under fluorescence microscope (Figure 5). In the wild type hyphae, cell nuclei were intensive and overlap, but in *Ssams2*-silenced transformants the cell nuclei were more dispersed. Both in hyphae and in germinating protoplasts, the numbers of cell nucleus in the same length of mycelium in *Ssams2*-silenced transformants were significantly less than that in the WT strain (Figure 6). The results demonstrated that *Ssams2* is required for chromosome segregation in *S. sclerotiorum*.

### *Ssams2* Is Involved in Histone Genes and Cell Cycle Genes Expression

**FIGURE 7:**
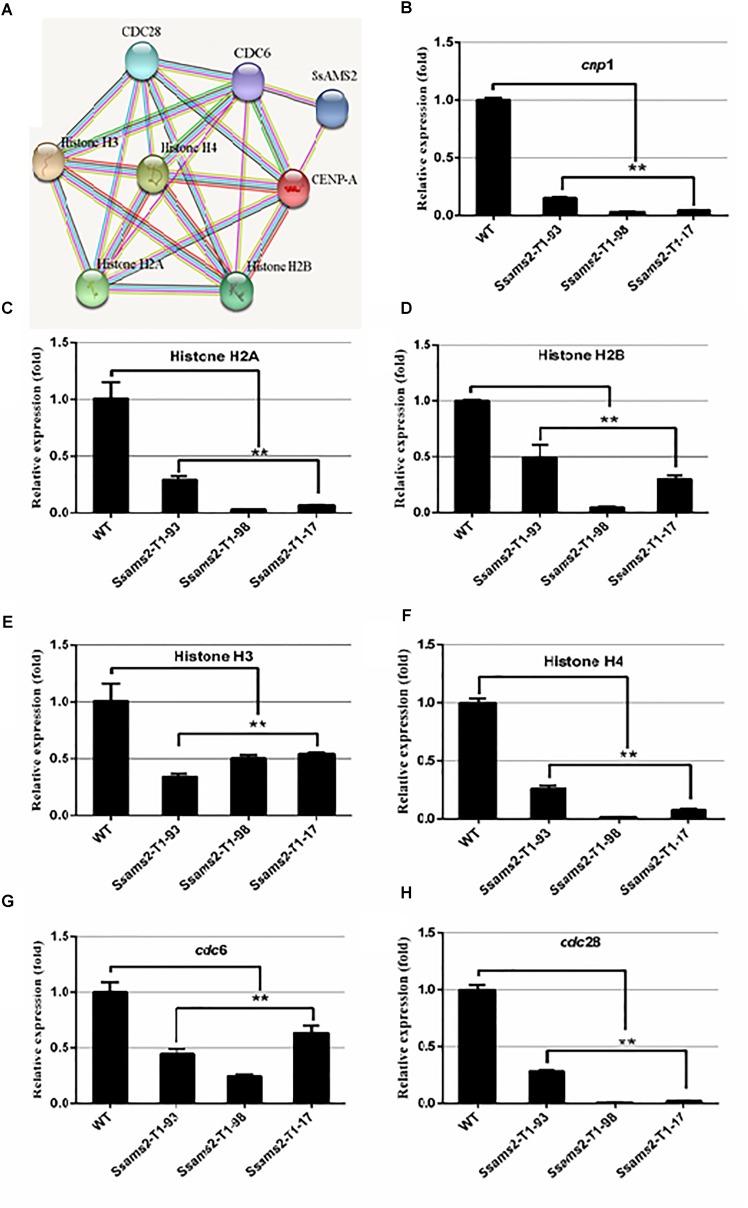
*Ssams2* is involved in the histones and cell cycle related genes transcription. **(A)** Function associate network of histone H2A, histone H2B gene, histone H3 gene, histone H4 gene, CENP-A, CDC6, CDC28 in *S. sclerotiorum* was predicted by STRING database. The known interactions lines in pieces of in purple and in cyan, the predicted interactions lines in red, blue, and green, others lines are in yellow, black, and lilac. **(B–H)** Histone H2A gene, histone H2B gene, histone H3 gene, histone H4 gene, CENP-A homolog gene *cnp1*, *cdc6*, and *cdc28* relative expression were monitored using real-time PCR (one-way ANOVA, ^∗∗^indicates significance at *p* < 0.01).

We have showed that reduced the expression of *Ssams2* could impaired chromosome segregation. Appropriate levels of histone expression are critical for DNA replication and chromosome segregation (Gunjan et al., 2005). *ams2* of *S. pombe* which is homologous with *Ssams2* has been demonstrated to be essential for histone genes transcription (Takayama and Takahashi, 2007). The histones (H2A, H2B, H3, H4, CENP-A in *S. sclerotiorum*), cell cycle related proteins (CDC6 and CDC28 in *S. sclerotiorum*) and SsAMS2 were analyzed using STRING database^1^ to derive the protein–protein interaction (PPI) network (Hsia et al., 2015) and the potential signaling pathways, SsAMS2 is directly related to SsCDC6 and SsCENP-A (Figure 7A). The expression levels of the cell cycle related gene *Sscdc6* (SS1G_04516), *Sscdc28* (SS1G_02296), histone genes H2A (SS1G_10959), H2B (SS1G_10960), H3 (SS1G_09608), H4 (SS1G_09609), and *Sscpn1* (SS1G_00304) in the RNAi transformants were down-regulated significantly (Figures 7B–H). These data demonstrate that chromosome segregation was disturbed in *Ssams2*-silenced strains, the transcription level of histone genes and cell cycle genes also were impaired.

## Discussion

In *S. pombe*, AMS2 as GATA transcriptional factor (TF) could activate histone genes transcription. After overproduced AMS2, chromosome instability and all the core histone genes were augmented expression (Takayama et al., 2010). The *ams2* gene is required for centromere function (Chen et al., 2003), destruction of *ams2* can help to prevent core histone genes expression. AMS2 stability expression is also required to ensure the coupling of mitosis to DNA replication (Trickey et al., 2013). So, whatever the AMS2 overproduction or absence, the defects in global cell division, histone genes expression, central centromere and chromosome stability can occur(Takayama and Takahashi, 2007; Takayama et al., 2010). In *ams2* mutant cells, the total amount of SpCENP-A deposition in the central centromeres was decreased compared to in the wild-type controls and showed missegregation of chromosome (Takahashi et al., 2005). The colony formation of fission yeast was exceedingly slow after the *ams2* gene was deleted and displayed abnormal mitosis accompany the aberrant chromosomes segregation (Chen et al., 2003). GATA TFs are broadly distributed in eukaryotes, fungal GATA factors have been demonstrate to be associate with diverse functions such as nutrient signal response, mating type switch, nitrogen control, siderophore biosynthesis, light regulated photomorphogenesis, circadian regulation, developmental differentiation, and morphogenesis (Tsang et al., 1997; Perlman et al., 1998; Charron et al., 1999; Reyes et al., 2004). In this study, SsAMS2 as a GATA- box contains protein homologous to the AMS2 in *S. pombe* (Figure 1). Based on the roles of AMS2 orthologs, we speculated that SsAMS2 may be involved in fungal growth, development and chromosome segregation in *S. sclerotiorum*. As a method to reduce the transcript accumulation, RNAi strategy was used to investigate the function of *Ssams2*. Compared with the wild-type strain, the *Ssams2*-silenced strains slowed hyphal growth and development, produced hyphae with wider angled branches and damaged the formation of infection cushion (compound appressoria) (Figures 2, 3).

*Sclerotinia sclerotiorum*, an aggressive phytopathogen, infects hundreds of species of plant and cause huge economic damage (Guyon et al., 2014; Yang et al., 2018). As a host-non-specific fungus, the *S. sclerotiorum* infection process has evolved more sophisticated and comprehensive (Lyu et al., 2016; Liu et al., 2018). Recently, a new infection mechanism, two-phase compatibility model by *S. sclerotiorum* was proposed. At first stage, the pathogen avoids host basal defense reactions and evades recognition. During this stage, the compound appressoria can penetrate cuticle but cannot penetrate the plant cell walls, the pathogen involves a transient biotrophic interaction stage (Kabbage et al., 2015; Liang and Rollins, 2018). Following this stage, mycelium which grows within the apoplastic space secrete OA, cell wall degrading enzymes and other pathogenicity factors, kill the plant cells and the fungus switch to necrotrophic growth (Kabbage et al., 2015; Liang and Rollins, 2018).

Compound appressoria are hyphal tip-differentiated multicellular infection structures formed by many plant-pathogenic fungi on the host surface (Hofman and Jongebloed, 1988; Boenisch and Schäfer, 2011). In *S. sclerotiorum*, impenetrable surfaces, such as dialysis tubing, parafilm, plastics, glass, and cellophane are often efficiently triggering compound appressoria differentiation (Li et al., 2018). Compound appressoria is critical for penetrating into the host cells and the complexity of compound appressorium is relevant to physical resistance to penetration on the host surface (Tariq and Jeffries, 1984; Jamaux et al., 2007; Huang et al., 2008). The penetration pegs breach the cuticle layer, bulbous and multi-lobed subcuticular vesicles form and subcuticular infection hyphae were produced which spread horizontally beneath the cuticle to comprise the leading colonization front (Li et al., 2012; Liang and Rollins, 2018). The deletion of the genes associated with compound appressoria formation leads to defect in appressorium formation and infection initiates (Liang et al., 2015b). For example, *Sscaf1* (compound appressorium formation related gene 1), which encodes a secretory protein and is required to infection cushions formation, plays a key role in host penetration (Xiao et al., 2014). Likewise, *Ssggt1* (γ-glutamyl transpeptidase) regulate substrates GSH/GSSG balance, and function in compound appressorium development, *Ssggt1* mutants revealed a decrease in infection efficiency (Li et al., 2012). In this study, WT strain rapidly differentiated infection cushions from vegetative hyphae on contact with glass slide. However, very few infection cushions were differentiated by the *Ssams2*-silenced strains (Figure 3B). The RNAi and WT strains were inoculated on common bean leaves, the *Ssams*2-silenced strains showed significant virulence defects (Figure 4).

Additionally, OA as a virulence factor has been demonstrated by studies, the full virulence requires a dynamic control of OA accumulation through multiple mechanisms (Liang and Rollins, 2018). OA accumulation lowers the ambient pH and the production of OA is critical for host colonization (Cessna et al., 2000; Rollins and Dickman, 2001; Davidson et al., 2016). *Ssrhs1*-silenced strains showed a defect in compound appressoria formation, OA secreted decreased and virulence defect (Yu et al., 2017). The *Ssnox1* mutant exhibited reduced efficiency in oxalate production, attenuated virulence (Kim et al., 2011). In this study, previous results show that *Ssams2* play an important role in hyphae growth, we hypothesize that the slowed mycelium growth caused the defect virulence of RNAi strains. The assayed of OA production on PDA supplemented with BPB showed that the less OA secreted could not solely attribute to the slowed mycelium growth (Figure 3D). The secreted levels of OA in *Ssams2*-silenced strains were lower than that in WT strain (Figure 3D and Supplementary Figure S5). The leaves which wounded prior to infection was used, showed partially rescue in infection efficiency compared with the healthy host. From the above, in *S. sclerotiorum*, the weaken of virulence might be associated with the growth and development of mycelium, compound appressoria formation, and OA secreted rather than a single factor. These results demonstrate that *Ssams2* involved in hyphae growth, compound appressorium formation and OA secreted, these factors common leading to failure in host penetration, attenuated virulence.

In eukaryotic cells, the two of each of the H2A, H2B, H3, and H4 comprise the nucleosome which is the basic unit of chromatin (Zhou et al., 2015; Takayama et al., 2016). The core histone genes showed evolutionarily conserved organization and appropriate transcription levels are essential for DNA damages repaired, chromosome segregation, mitosis, spermatogenesis, and cell viability (Osley, 1991; Maruyama et al., 2006; Marzluff, 2010; Takayama et al., 2016; Franciosi et al., 2017). AMS2 binds to the AACCT-box which exist in all core histone gene promoters via the zinc finger motif (Takayama et al., 2016), which is critical for activating histone genes transcription with Teb1 in cell cycle S phrase (Valente et al., 2013). Cell cycle-dependent *ams2* levels fluctuating manner is crucial for the centromere nucleosome formation and genetic stability (Takayama et al., 2010; Trickey et al., 2013). In the *ams2* mutants, the cell cycle was disturbed and the process of transcriptional oscillation of histone genes was disappeared (Takahashi et al., 2005). On the other hands, accompany DNA replication, the cell-cycle-dependent accumulation of histone mRNAs was markedly suppressed (Takayama and Takahashi, 2007). In both yeast and higher eukaryotes, the DNA replication was interfered with by genotoxic agents which impaired histone gene expression (Han et al., 1987; Nelson et al., 2002; Gunjan et al., 2005). In yeast, a mutant form of CDC6 that lacked its enzyme activity was unable to copy their DNA and the cell did not divide (Chang et al., 2015). Kinetochore is indispensable for chromosome segregation and CENP-A as a centromere-specific histone H3 variant plays an important role for the formation of kinetochore (Chen et al., 2003). AMS2 GATA factor which promotes histone gene activation is required for CENP-A deposition (Takayama et al., 2008). PPI network obtained from STRING database showed that SsAMS2 interacts with SsCDC6 and SsCENP-A directly, implicating that SsAMS2 might involve in DNA replication and chromosome segregation in *S. sclerotiorum*. In this study, we found that the numbers of cell nucleus in *Ssams2*-silenced transformants were significantly reduced (Figure 6). It is likely that the less expression of *Sscnp1*, *Sscdc6* and histone genes is due to the reduced transcription accumulation of *Ssams2* by RNAi strategy (Figure 7). It is well defined that proper *Ssams2*, *Sscnp1* and core histone genes accumulation are vital for normal DNA replication and chromosome segregation.

In conclusion, the *Ssams2* gene is characterized to encode a GATA-type transcription factor orthologous to the *S. pombe ams2* gene which is required for efficient core histone genes and cell cycle genes transcription. This study provides evidence that *Ssams2* plays a critical role in the histone genes transcription and chromosome segregation. The *Ssams2*-silenced stains were also slowed hyphal growth, defective in compound appressorium formation, impaired OA secreted, and reduced virulence on host plants.

## Materials and Methods

### Fungal Strains and Culture Conditions and Plant Materials

The wild type isolate *S. sclerotiorum* “UF1” isolated from diseased petunia in Florida (Li et al., 2018) was used in this study. Strains were cultured, unless otherwise stated, on PDA medium at 25°C (Fan et al., 2017). *S. sclerotiorum* transformants were cultured on PDA amended with 100 μg/ml geneticin (Roche, Indianapolis, IN, United States) (Liu et al., 2018) and 50 μg/ml bromophenol blue (Sigma-Aldrich, United States) (Li et al., 2018). Liquid YPSU medium (50 ml containing yeast extract 4g, K_2_HPO_4_ 1g, Mg_2_SO_4_ ⋅ 7H_2_O 0.5g, sucrose 15g, pH 6.5) was used to measure pH (Rollins, 2003). Common bean (*Phaseolus vulgaris*) used for pathogenicity assay grew in the greenhouse.

### Bioinformatics Analysis of SsAMS2

In the *S. sclerotiorum* genome, nine GATA-box-containing proteins were found based on PFAM analysis (Li et al., 2017; Liu et al., 2018) and a protein (SS1G_03252) was choosed for this study. Using the MEGA software to constructed the phylogenetic tree (Liu et al., 2018). All sequences available for *Ssams2* homologs form *B. cinerea*, *M. coronariae*, *M. brunneum*, *M. majus*, *C. fioriniae*, *A. flavus*, *A. bombycis*, *T. marneffei* and *S. pombe*. SsAMS2 (XP_001595163.1), *D. rerio* GATA-4 (NP_571311.2), *M. musculus* GATA-4 (NP_001297539.1), *H. sapiens* GATA-4 (NP_001295022.1), *D. melanogaster* (NP_001262620.1), and *S. pombe* AMS2 (NP_588400.2) was performed using BlastSMART service (Liu et al., 2018).

### Generation of RNAi Constructs and Transformation

The *Ssams2* gene RNA-silencing vector construction strategy was described previously (Nguyen et al., 2008; Fan et al., 2017; Liu et al., 2018). The Target 1 (254 bp) and Target 2 (352 bp) fragments with *Hin*dIII restriction enzyme site were amplified with the primers pSD1-T1-F/R and pSD1-T2-F/R (Supplementary Table S1). The successfully constructed plasmids used for PEG-mediated protoplasts transformation (Rollins, 2003). Transformants were cultured and purified as described previously (Fan et al., 2017). Morphological characteristics analysis and hyphal growth rate observation. Sclerotia culture and collection, colony diameters, hyphal morphology of each strain were measured or observed as described previously (Liu et al., 2018).

### Virulence Assays

Seven-weeks-old common bean leaves were used as described previously (Rollins, 2003) and inoculated the mycelium plug (5 mm diameter) and incubated in proper environment as described previously (Qu et al., 2014; Wang et al., 2016). For the wounded treatment, a cut was made through the adaxial and abaxial surface of each leaves with a needle before inoculation. The photographs were taken with a Sony ILCE 6000. The lesion areas were calculated by tracing the necrotic area (Li et al., 2012). The experiments were performed at least three times.

### The Analysis of Gene Transcription Accumulation by qRT-PCR

The genes expression was analyzed by qRT-PCR. Total RNA was isolated using TRIzol reagent according to the manufacturer’s instructions (Invitrogen, Carlsbad, CA, United States) (Fan et al., 2017) and the specific methods refer to previous studies (Schmittgen et al., 2000; Gutierrez et al., 2008; Liu et al., 2018).

### Hyphae Cell Nuclei Numbers Assay

Hyphae-plugs of WT strain and *Ssams*2-silenced strains were inoculated on PDA overlaid with cellophane 16 h. The hyphae were stained in 4′,6-diamidino-2-phenylindole (DAPI) in the dark for 30 min (Takayama et al., 2008). Protoplasts of *Ssams*2-silenced transformants and wild type strain were prepared (Rollins, 2003) and incubated onto cellophane-overlaid PDA plates, the germinating hyphae were stained by DAPI. The same length of hyphae cell nuclei was observed and counted in the fluorescence microscope (Nikon ECLIPSE Ts2R).

## Author Contributions

LL, QW, and HP planned and designed the research. LL and HP wrote the manuscript. All authors performed the experiments and analyzed the data.

## Conflict of Interest Statement

The authors declare that the research was conducted in the absence of any commercial or financial relationships that could be construed as a potential conflict of interest.

## Footnotes

1 https://string-db.org/

## Supplementary Material

Figure S1 Expression patterns of *Ssams2.* Relative expression of *Ssams2* at different developmental stages (hyphae, sclerotia, and apothecium). The abundance of cDNA from hyphae was assigned a value of 1. Gene expression values are normalized to that of the actin transcripts. Values are the means ± SD (*n* = 3). Asterisks indicate significant differences compared with the expression of *Ssams2* in hyphae (^∗∗^*P* < 0.01, one-way ANOVA).

Figure S2 Sequence analysis of *Ssams2* in *S. sclerotiorum.* BLASTN search was used to show low nucleotide sequence similarities between *Ssams2* and others genes in *S. sclerotiorum* genome.

Figure S3 Identification of geneticin resistance genes by PCR. Conventional PCR were performed for geneticin resistance genes and produced 650 bp PCR products from RNAi strains. M, DNA molecular size marker; WT, wild type strain; lanes 1–3: putative transformants *Ssams2*-T1-93, *Ssams2*-T1-98, and *Ssams2*-T1-17; lanes 4–6: putative transformants *Ssams2*-T2-202, *Ssams2*-T2-102, and *Ssams2*-T2-108.

Figure S4 Effect of *Ssams2* silencing on sclerotium development in culture. **(A)** Comparison of sclerotia morphology and size among the WT and the silenced transformants. **(B)** Average sclerotium dry weight of the WT strains and the RNA-silenced transformants. **(C)** Average sclerotium numbers of the WT and the *Ssams2* silencing strains. WT and selected RNAi-silenced transformants were cultured on 9 cm PDA culture plates at 25°C. Each data point represents the means ± SD from three biological repeats (*n* = 3).

Figure S5 The pH was measured by accurate range pH test paper. WT strains and the RNA-silenced transformants were inoculated in YPSU culture and incubate at 25°C for 24 h (WT) and 48 h (RNAi), make the clones have the same length of diameter.

Table S1 Primers used in this study.
