# Supplementary figures and images for: Ssams2, a Gene Encoding GATA Transcription Factor, Is Required for Appressoria Formation and Chromosome Segregation in Sclerotinia sclerotiorum
Source: Front Microbiol. 2018 Dec 6;9:3031. doi: 10.3389/fmicb.2018.03031 (PMC6291475; doi:10.3389/fmicb.2018.03031)

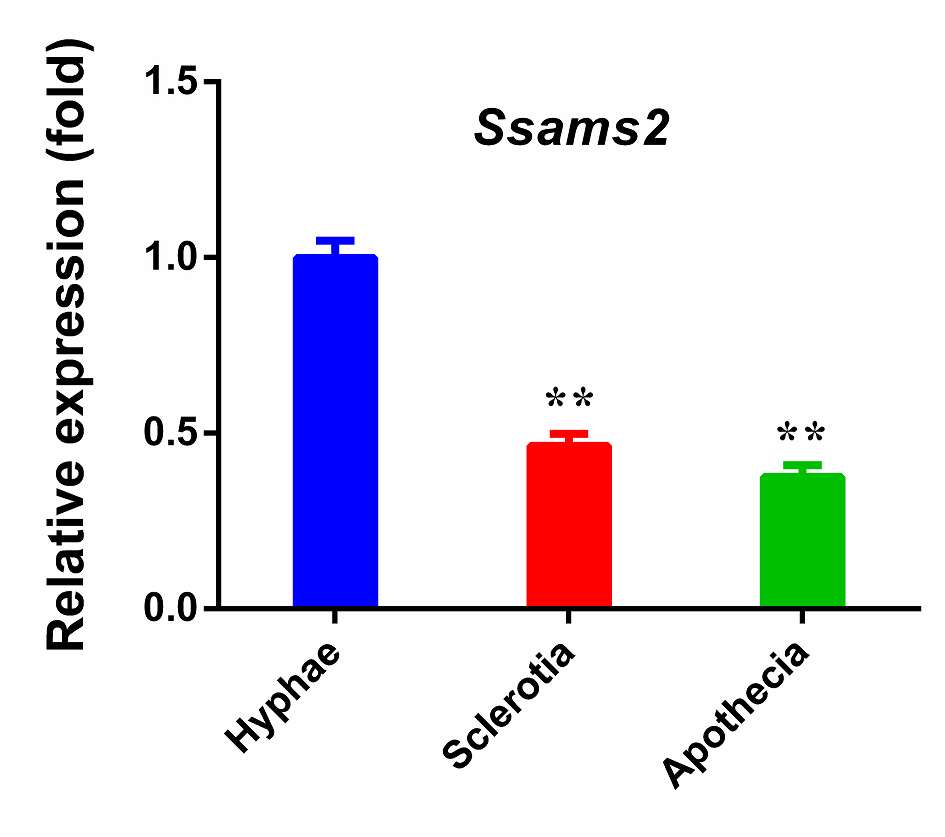

Supplement: Figure S1 — Expression patterns of Ssams2. Relative expression of Ssams2 at different developmental stages (hyphae, sclerotia, and apothecium). The abundance of cDNA from hyphae was assigned a value of 1. Gene expression values are normalized to that of the actin transcripts. Values are the means ± SD (n = 3). Asterisks indicate significant differences compared with the expression of Ssams2 in hyphae (∗∗P < 0.01, one-way ANOVA). [file Image_1.TIF]

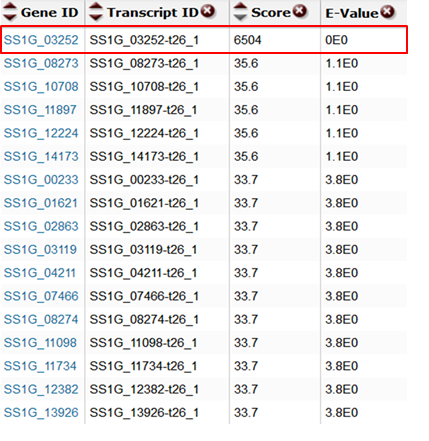

Supplement: Figure S2 — Sequence analysis of Ssams2 in S. sclerotiorum. BLASTN search was used to show low nucleotide sequence similarities between Ssams2 and others genes in S. sclerotiorum genome. [file Image_2.TIF]

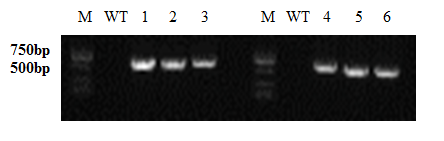

Supplement: Figure S3 — Identification of geneticin resistance genes by PCR. Conventional PCR were performed for geneticin resistance genes and produced 650 bp PCR products from RNAi strains. M, DNA molecular size marker; WT, wild type strain; lanes 1–3: putative transformants Ssams2-T1-93, Ssams2-T1-98, and Ssams2-T1-17; lanes 4–6: putative transformants Ssams2-T2-202, Ssams2-T2-102, and Ssams2-T2-108. [file Image_3.TIF]

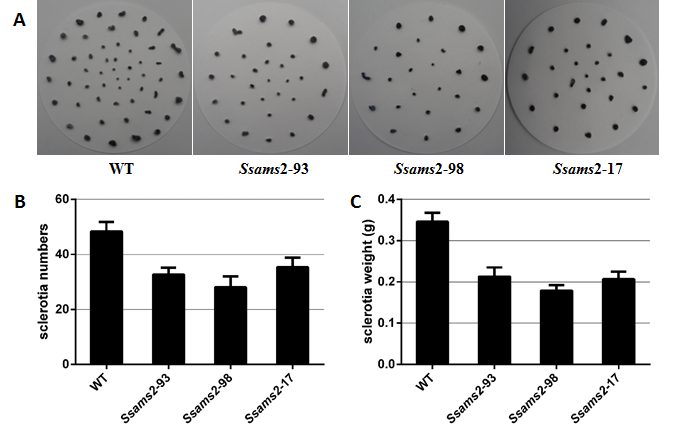

Supplement: Figure S4 — Effect of Ssams2 silencing on sclerotium development in culture. (A) Comparison of sclerotia morphology and size among the WT and the silenced transformants. (B) Average sclerotium dry weight of the WT strains and the RNA-silenced transformants. (C) Average sclerotium numbers of the WT and the Ssams2 silencing strains. WT and selected RNAi-silenced transformants were cultured on 9 cm PDA culture plates at 25°C. Each data point represents the means ± SD from three biological repeats (n = 3). [file Image_4.TIF]

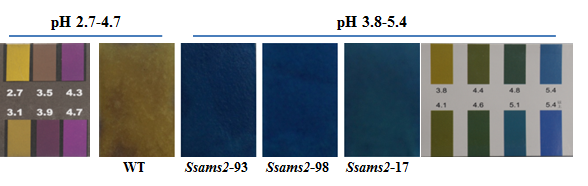

Supplement: Figure S5 — The pH was measured by accurate range pH test paper. WT strains and the RNA-silenced transformants were inoculated in YPSU culture and incubate at 25°C for 24 h (WT) and 48 h (RNAi), make the clones have the same length of diameter. [file Image_5.TIF]
