# Supplementary material for: Ssams2, a Gene Encoding GATA Transcription Factor, Is Required for Appressoria Formation and Chromosome Segregation in Sclerotinia sclerotiorum
Source: Front Microbiol. 2018 Dec 6;9:3031. doi: 10.3389/fmicb.2018.03031 (PMC6291475; doi:10.3389/fmicb.2018.03031)
Supplement: Table S1 — Primers used in this study. [file Table_1.DOCX]

**Table S1. Primers used in this study.**

|  | **Primer** | **Primer sequence** |
| --- | --- | --- |
| RNAi Primers (*Hin*dIII) | pSD1-T1-F | CCCAAGCTTACGCAATGACCTCAGATTC |
|  | pSD1-T1-R | CCCAAGCTTCTCCTTGTTCATCGTTCCA |
|  | pSD1-T2-F | CCCAAGCTTCTCAAACAGTTCCACCAATG |
|  | pSD1-T2-R | CCCAAGCTTCGTCTTCCCCTCTTCACA |
| Geneticin  gene Primers | Gene-F | TGTCCGGTGCCCTGAATGAACT |
|  | Gene-R | GCCGCCAAGCTCTTCAGCAATAT |
| qRT-PCR  Housekeeping genes Primers | Actin-F | GAATGTGTAAGGCCGGTTTCGC |
|  | Actin-R | CATCCCAGTTGGTGACGACACC |
|  | Tubulin-F | GGTGAGCATGGTCTTGACGG |
|  | Tubulin-R | CCCTCAGCCTCACGACGAAC |
| qRT-PCR Primers | *Ssams2*-QF | CCACCTCGTATCTCCTCAT |
|  | *Ssams2*-QR | CATCCCAGTTGGTGACGACACC |
|  | HistoneH2AQF | TTCGCAATGTGTTCTCAATC |
|  | HistoneH2AQR | ATGACTCACCAGCACCAA |
|  | HistoneH2BQF | GGAGACTTACTCTTCATACATC |
|  | HistoneH2BQR | TGGACTTGCTTGAGAACTAT |
|  | HistoneH3QF | GGCTCGTACCAAGCAAACTG |
|  | HistoneH3QR | GAAGTCTTGGGCGATTTCAC |
|  | HistoneH4QF | CTCTCACCGTCATTCAGTT |
|  | HistoneH4QR | TTATGCCTTGGATGTTGTCT |
|  | *cnp1*QF | TATCGCTGGAAGAAGAATCA |
|  | *cnp1*QF | GCTTACCTCTAACGGCAAT |
|  | *cdc*6QF | CGATGCCGATTCTATGGA |
|  | *cdc*6QR | GTTCTTGGTAATGCGATTCT |
|  | *cdc*28QF | CGAGGCTGAAGATGAAGG |
|  | *cdc*28QR | CGTGAACGATATTAAGGAGTC |
